# Lofgren Syndrome in a 35‐Year‐Old Female: A Case Report

**DOI:** 10.1002/ccr3.71185

**Published:** 2025-10-14

**Authors:** Abel Tenaw Tasamma, Sebhatleab Teju Mulate, Etsegenet Yayut Munie, Kasim Arga Berkaga, Abdulrahim Mehadi, Nebiyu Getachew Mekonnen

**Affiliations:** ^1^ School of Medicine College of Health Sciences, Addis Ababa University Addis Ababa Ethiopia; ^2^ John H. Stroger Jr. Hospital of Cook County Chicago Illinois USA

**Keywords:** erythema nodosum, Ethiopia, Löfgren syndrome, sarcoidosis

## Abstract

Lofgren syndrome is an acute and pathognomonic form of sarcoidosis characterized by fever, hilar lymphadenopathy, arthritis, and erythema nodosum (EN). We report a 35‐year‐old female patient from Ethiopia who presented with a 4‐day history of arthritis involving bilateral ankles, knees, and wrists associated with fever and skin lesions typical of EN. Chest imaging (X‐ray and computed tomography (CT) scan) revealed bilateral hilar and mediastinal lymphadenopathy as well as perilymphatic nodules in the mid and upper zones of both lungs. Workup for alternative diagnoses, including tuberculosis, was negative. After the diagnosis of Lofgren syndrome was made, she was started on a low dose of prednisolone, which resulted in a prompt and marked improvement in her symptoms.

**Summary**

- Löfgren syndrome should be included in the differential diagnosis for a young patient presenting with arthritis, fever, and skin lesions resembling erythema nodosum (EN).

## 1 Introduction

Sarcoidosis, a multi‐system granulomatous disease that predominately affects the lungs and skin [1]. While its exact underlying etiology remains poorly understood, there is some literature that shows that the disease is caused by an aberrant host response to various environmental antigens or infectious agents [2, 3]. Sarcoidosis runs a chronic course; however, a subset of patients may present with an acute onset of fever, hilar lymphadenopathy, arthritis, and erythema nodosum (EN) in different proportions, a constellation of manifestations referred to as Lofgren syndrome [4, 5, 6]. It exhibits a strong female predominance, with peak incidence occurring between the third and fourth decades of life. Studies reveal the highest prevalence among individuals of Northern European ancestry—particularly Scandinavian populations—while remaining relatively uncommon in regions such as Africa and Japan [7, 8, 9]. Lofgren syndrome is often self‐limiting, with most cutaneous manifestations resolving spontaneously [1] Nonetheless, joint symptoms may persist chronically, and some patients experience recurrent episodes [10]. The diagnosis of Lofgren syndrome is made based on classic presenting symptoms [11]. We hereby present a case of sarcoidosis with Lofgren syndrome in a 35‐year‐old female patient from Ethiopia. Currently, there are only a few reports of Lofgren syndrome from Africa, and to the best of our knowledge, this is the first case report from Ethiopia.

## 2 Case History

### 2.1 Initial Presentation

A 35‐year‐old hypertensive female, under treatment with hydrochlorothiazide 25 mg and amlodipine 5 mg daily for the past 9 months, presented with a four‐day history of joint pain and swelling affecting bilateral ankles, knees, and wrists, accompanied by a rash on the lower extremities and forearms, and a low‐grade intermittent fever. She reported a three‐month history of intermittent productive cough, temporarily relieved by repeated seven‐day courses of amoxicillin, loss of appetite, and fatigue. Previously, she was diagnosed with genital tuberculosis, undergoing unspecified surgical intervention and completing a six‐month course of first‐line antituberculous treatment. There was no pertinent history suggesting similar clinical history in her family members.

**FIGURE 1:**
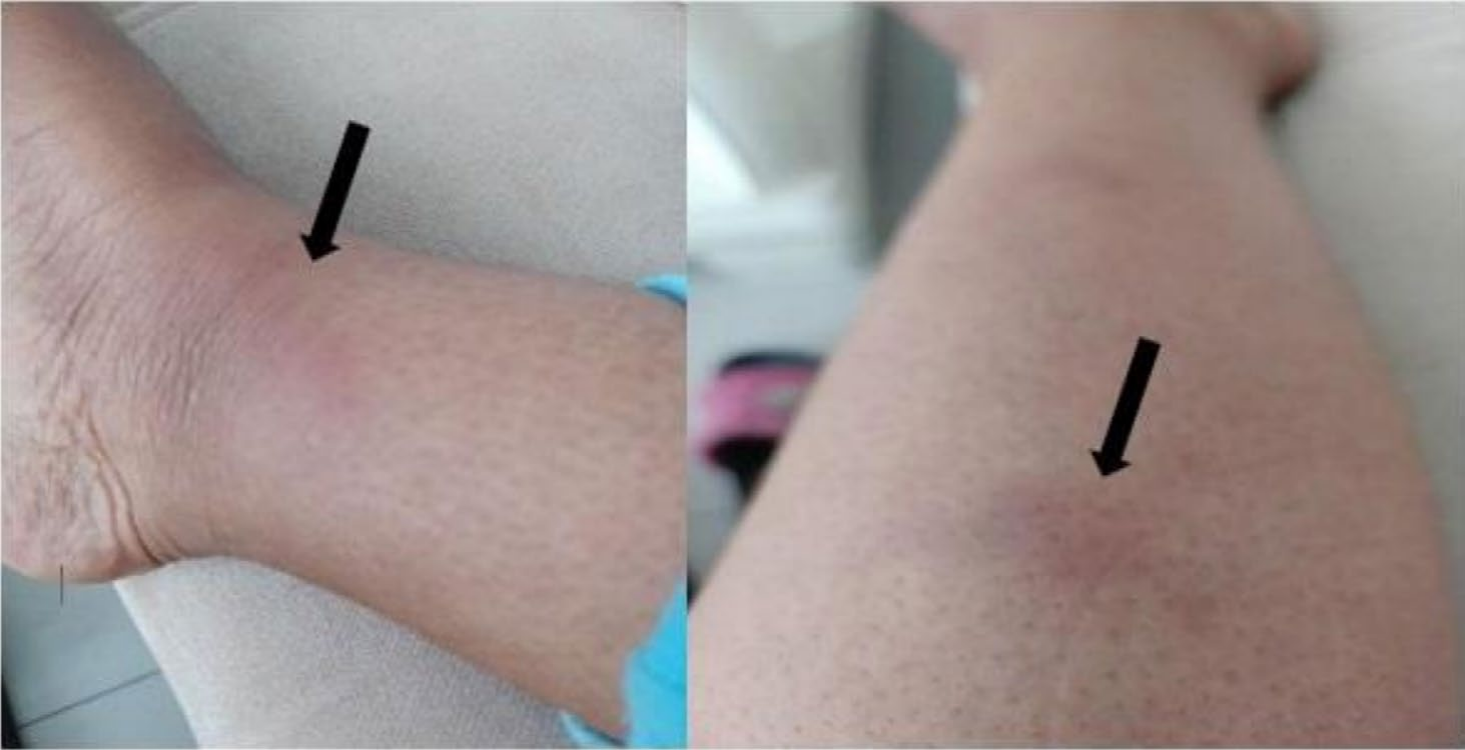
Multiple erythematous nodules over the leg (erythema nodosum).

**FIGURE 2:**
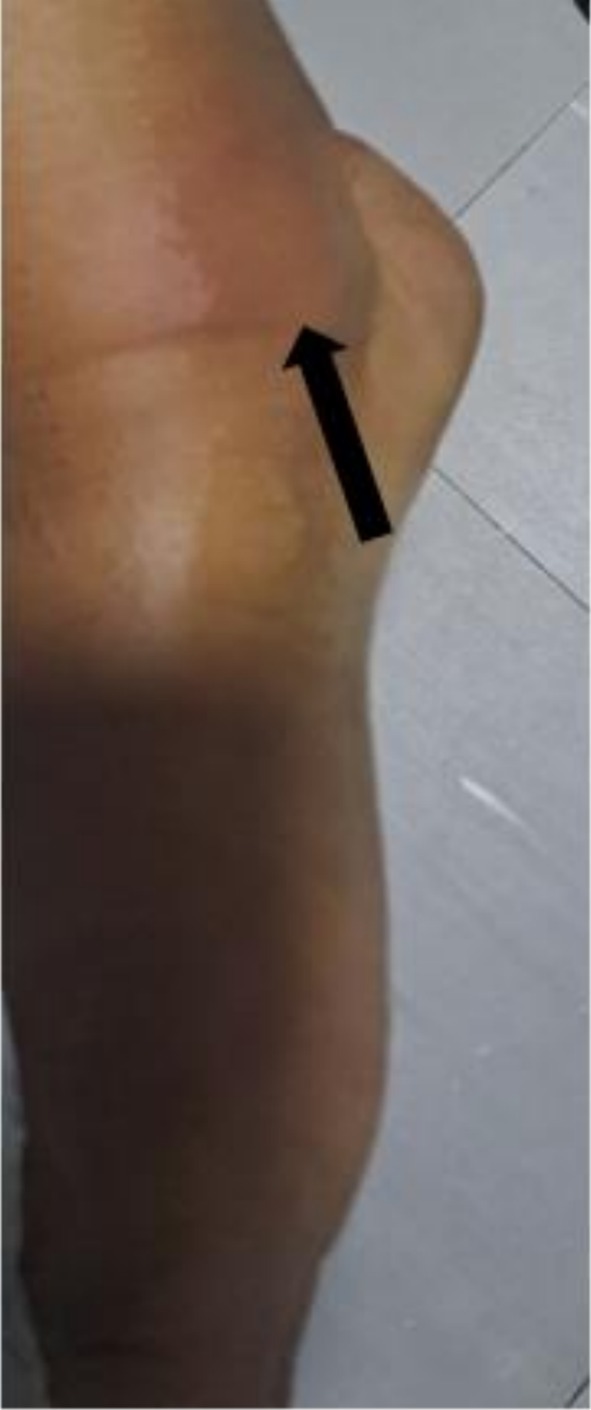
A swollen and erythematous right ankle joint (arrow).

Initial physical examination revealed hypertension (160/100 mmHg), tachycardia (112 beats/min), a respiratory rate of 24 breaths/min, body temperature of 36.9°C, and oxygen saturation of 98% on room air. No significant lymphadenopathy was detected. She exhibited multiple tender erythematous nodules on her bilateral shins and forearms, indicative of erythema nodosum (Figure 1). Musculoskeletal examination showed swollen, erythematous, and tender bilateral ankle (Figure 2) and knee joints.

### 2.2 Differential Diagnosis

The most important differential diagnosis that was considered includes reactive arthritis, connective tissue disease associated arthritis, and viral arthritis.

### 2.3 Investigations and Treatment

**FIGURE 3:**
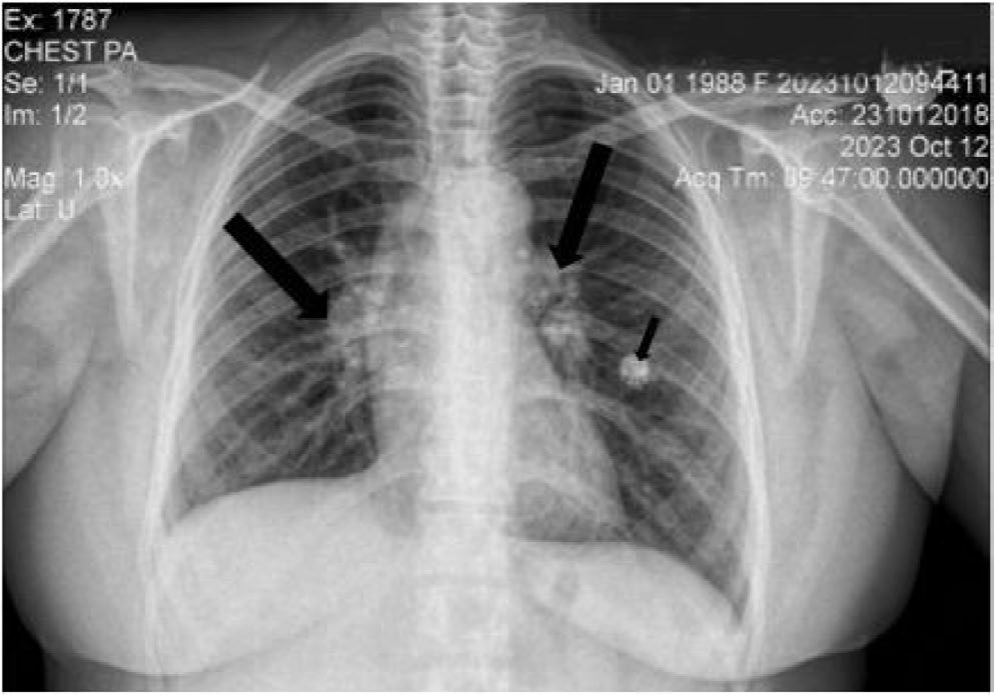
Bilateral hilar lymphadenopathy (large arrows) and a calcified nodule (small arrow) on chest X‐ray.

**FIGURE 4:**
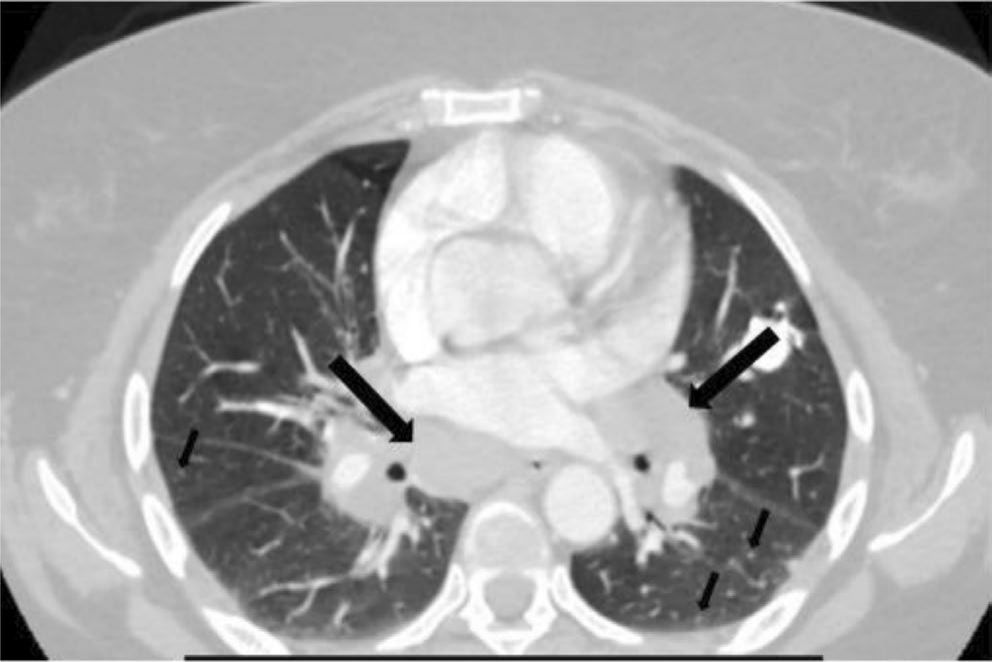
Bilateral hilar lymphadenopathy (big arrows) and multiple perilymphatic nodules (small arrows) on chest CT scan.

Laboratory investigations revealed a hemoglobin level of 16 mg/dL, a normal total leucocyte count, and platelets. Erythrocyte sedimentation rate (ESR) and C‐reactive protein (CRP) levels were elevated at 50 mm/h and 69 mg/L, respectively. Renal and liver function tests, serum electrolytes, thyroid function, fasting blood sugar, vitamin D, and calcium levels were all within normal limits. Urinalysis was unremarkable. Lipid profile showed total cholesterol of 254 mg/dL, triglycerides of 152 mg/dL, high‐density lipoprotein (HDL) of 53 mg/dL, and low‐density lipoprotein (LDL) of 171 mg/dL. Human immunodeficiency virus (HIV), hepatitis B virus (HBV), and hepatitis C virus (HCV) serologies were all negative, and antinuclear antibody (ANA) titer was 1:100. Chest X‐ray revealed bilateral hilar lymphadenopathy with left mid‐lung zone calcified nodules (Figure 3). ECG was unremarkable. Contrast‐enhanced computed tomography (CT) scan of the chest showed bilateral hilar and mediastinal lymphadenopathy, perilymphatic nodules in the mid and upper zones of both lungs, and a calcified nodule in the left upper lobe (Figure 4). Sputum examination using Gene‐Xpert MTB/RIF test didn't detect *Mycobacterium tuberculosis* . A foot X‐ray indicated an old fracture at the base of the 3rd metatarsal with periarticular osteopenia.

The diagnosis of sarcoidosis with Lofgren syndrome was established based on clinical presentation (fever, arthritis, and EN) and imaging findings (bilateral hilar and mediastinal lymphadenopathy, and perilymphatic nodules in mid and upper lung zones on post‐contrast CT). Initial treatment with oral diclofenac 75 mg as needed was ineffective. Subsequently, prednisolone (30 mg/day) was administered.

### 2.4 Outcome and Follow‐Up

The patient had resolution of joint pain and skin rash within 2 weeks and a decrease in ESR to 30 mm/h. The prednisolone dose was tapered to 10 mg/day over 2 weeks, resulting in significant improvement in skin lesions and joint swelling, and resolution of pulmonary symptoms. The patient was referred for pulmonology follow‐up.

## 3 Discussion

Our patient exhibited all four features of Lofgren syndrome, that is, bilateral hilar lymphadenopathy, fever, EN, and arthritis. Moreover, the fact that she had a prompt response to a short course of low‐dose prednisolone further substantiates the diagnosis. Lofgren syndrome, a peculiar and pathognomonic manifestation of sarcoidosis, presents acutely over days to weeks and is observed in 5%–10% of patients [12]. Its specificity to sarcoidosis is such that its identification often obviates the need for histopathological confirmation, provided that other potential causes are conclusively excluded [13].

Erythema nodosum presents as an erythematous and tender nodule over the anterior shins and is more commonly seen in females [1]. It is imperative to note that this lesion should not be biopsied as the associated histologic finding (panniculitis) is not specific enough to make a diagnosis of sarcoidosis [14]. The arthritis associated with Lofgren syndrome typically involves the ankles bilaterally; although other large joints such as the knees, elbows, and wrists could also be affected [15]. This manifestation of sarcoidosis is reported to occur predominantly in men [1]. Fever, which is more commonly seen in females, and hilar adenopathy, which is typically bilateral, complete the four cardinal components of Lofgren syndrome.

Even though the detection of Lofgren syndrome, which has a specificity of 95% to diagnose sarcoidosis [11], is made on clinical grounds, supportive laboratory tests such as ESR and angiotensin‐converting enzyme (ACE) should also be obtained. Lofgren syndrome is generally a self‐limiting condition with an excellent prognosis. Most patients show improvement with supportive care and non‐steroidal anti‐inflammatory drugs (NSAIDs), and the use of corticosteroids is infrequently necessary [2].

Richard et al. described a case of a 39‐year‐old Nigerian woman diagnosed with Lofgren syndrome after she presented with a two‐month history of dry and persistent cough, easy fatigability, and painful swelling of the ankles. Physical examination revealed hyper‐pigmented patches of early lupus pernio and tender erythema nodosum on her feet. Chest X‐ray features, serum ACE level, and other lab parameters supported the diagnosis of Lofgren and she was started on steroids and azathioprine. This patient had a more protracted course and did not have fever or lymphadenopathy and had more diffuse joint involvement [16]. Another case report from West Africa reported a sporadic case of Lofgren syndrome after a middle‐aged man presented with symptoms and laboratory investigations very similar to our case and prompted physicians in Sub‐Saharan countries to have a high index of suspicion despite the rarity of Lofgren in the region [17].

## 4 Conclusion

Therefore, Lofgren syndrome should be considered in the differential diagnosis of a young patient presenting with arthritis, fever, and skin lesions resembling EN. The presence of bilateral hilar lymphadenopathy confirms the diagnosis. Even though Lofgren syndrome is rarely reported from Ethiopia and Africa in general, we prompt medical professionals to have a high index of suspicion as misdiagnosis can result in unnecessary medical interventions.

## Author Contributions

**Abel Tenaw Tasamma:** conceptualization, investigation, writing – original draft, writing – review and editing. **Sebhatleab Teju Mulate:** conceptualization, writing – original draft. **Etsegenet Yayut Munie:** writing – original draft. **Kasim Arga Berkaga:** conceptualization. **Abdulrahim Mehadi:** writing – review and editing. **Nebiyu Getachew Mekonnen:** conceptualization.

## Disclosure

The authors have nothing to report.

## Consent

The patient provided written informed consent for publication of the case report.

## Conflicts of Interest

The authors declare no conflicts of interest.

## Data Availability

Data sharing not applicable to this article as no datasets were generated or analyzed during the current study.
